# Ash Transformation during Fixed-Bed Co-combustion of Sewage Sludge and Agricultural Residues with a Focus on Phosphorus

**DOI:** 10.1021/acsomega.3c00415

**Published:** 2023-03-31

**Authors:** Joel Falk, Thomas Karl Hannl, Marcus Öhman, Ali Hedayati, Nils Skoglund

**Affiliations:** † Energy Engineering, Department of Engineering Sciences and Mathematics, Luleå University of Technology, SE-97187 Luleå, Sweden; ‡ Thermochemical Energy Conversion Laboratory, Department of Applied Physics and Electronics, Umeå University, SE-90187 Umeå, Sweden; § BEST−Bioenergy and Sustainable Technologies GmbH, Inffeldgasse 21b, AT-8010 Graz, Austria; ∥ Institute of Chemical, Environmental & Bioscience Engineering, TU Vienna, AT-1060 Vienna, Austria

## Abstract

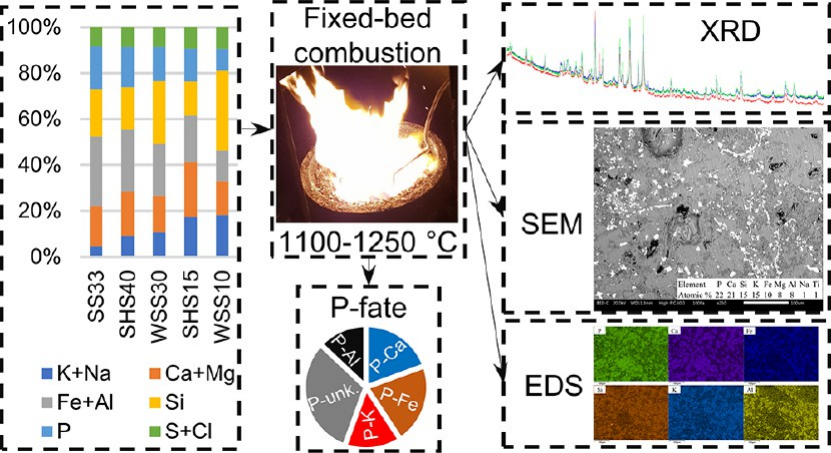

This work investigates the ash transformation during fixed-bed co-combustion of sewage sludge mixtures with the agricultural residues wheat straw and sunflower husks, focusing on the fate of phosphorus (P) in the resulting ash fractions. The study aims to determine suitable process parameters for fixed-bed combustion of fuels previously investigated in single-pellet experiments. The pure fuels and fuel mixtures were combusted in a 20 kW_th_ residential pellet burner while monitoring the flue gas composition, temperature, and particulate matter formation. Subsequently, the different ash fractions were collected and characterized by CHN, SEM/EDS, and XRD analysis. The results showed that co-combustion of sewage sludge and agricultural residues reduced the formation of particulate matter as well as the formation of slag. Co-combustion of sewage sludge with either agricultural residue resulted in a change in phosphate speciation, displaying higher shares of Ca and lower shares of Fe and Al in the formed orthophosphates as well as amorphous phases containing higher shares of K. The formation of K-bearing phosphates was hindered by the spatial association of P with Ca and Fe in the sewage sludge, the incorporation of available K in K-Al silicates, and the depletion of K in the P-rich melt phase. Compared to mono-combustion, co-combustion experiments showed the potential for improving the combustion performance and reducing the risk of slag formation. The outcome suggests that co-combustion is a feasible path to integrate waste streams in fixed-bed energy conversion with simultaneous formation of phosphates enabling P recovery.

## 1 Introduction

A reliable source of phosphorus (P) is a fundamental premise for human society as it is an irreplaceable nutrient required for all living things. On the basis of recent assessments, the current usage of P is unsustainable with the potential for severe consequences for human society at large.^1^ At the current usage, global P scarcity is a distinct possibility in the near future,^2^ which could pose a serious threat to the global food supply as modern agricultural systems depend on mineral P fertilizers.^3^ For this reason, significant research efforts are under way to find new ways to minimize the loss of P from societal waste streams to reduce our dependency on nonrenewable phosphate resources.^4,5^ Sewage sludge, a byproduct of municipal wastewater treatment plants, is a waste stream that has long been identified as suitable for P recovery. It is widely available in large quantities, has a high P content, and is produced in centralized facilities. Sewage sludge has been the subject of numerous investigations, with earlier research focused mostly on the disposal of sewage sludge.^6^ In recent years, the focus has shifted toward energy^7,8^ and P recovery.^9−12^

Direct use of sewage sludge as a fertilizer may be hazardous as it contains potentially toxic organic and inorganic compounds.^13−15^ Thermal conversion of sewage sludge is a suitable solution to this problem as it enhances the environmental compatibility of sewage sludge by breaking down harmful organic compounds^13^ and by immobilizing or separating toxic heavy metals.^16^ A downside with the thermal conversion of sewage sludge is that the resulting ash or char may have a low fertilizer value. The thermal process leads to the formation of mineral phosphate compounds that are not readily available to plants.^17−20^ Several postprocessing methods are currently being investigated to solve this issue.^12,21−23^

The underlying principle for the thermochemical postprocessing methods is to use additives in combination with a controlled temperature and atmosphere to alter the P species present in the ashes. Because conditions similar to these postprocessing methods can be achieved during the thermal conversion process, it is feasible that the targeted phosphate speciation could be achieved without the need for postprocessing. Recent studies have shown that the speciation of phosphorus in sewage sludge can be altered through co-converting sewage sludge with biomass or additives.^24−29^ In addition, the co-combustion of sludge with biomass has the potential for other auxiliary benefits. Co-combustion studies of sewage sludge with K- and Cl-rich biomass types such as wheat straw and waste-based residues have been shown to reduce the risk of fouling,^30^ agglomeration,^31^ sintering,^32^ and corrosion.^33,34^ Furthermore, the environmentally critical aspect of heavy metal concentration in ashes and its derived fertilizers could be somewhat controlled through co-combustion by separation and immobilization of these elements in the thermal process.^35,36^ As sewage sludge is characterized by a low heating value due to its inherent properties (moisture content, ash content), drying is often a necessary pretreatment step for the thermal conversion of sewage sludge.^11^ Thus, several positive synergies exist for co-combusting sewage sludge with relatively dry but challenging biomass residues with relatively low ash content.

P in sewage sludge ashes is mainly associated with Fe, Al, and Ca^19^ and is linked to poor agronomic performance.^20^ However, it has been shown that the agronomic performance of P in such ashes can be greatly improved by thermal treatment with alkali sulfates, which alters the speciation of P toward more plant available (K, Na)(Ca, Mg)PO_4_ phases.^17,21,37,38^ Thus, when designing fuel mixes of biomass and sewage sludge, the formation of K-bearing phosphates such as CaKPO_4_ and KMgPO_4_ is likely a suitable target to improve the P recovery potential of the ashes for both direct application and leaching.

Previous studies have shown that it is possible to target K-bearing phosphates by combustion or gasification of sewage sludge mixed with K-rich biomass.^27,28,39^ However, a substantial share of phosphates was still associated with Ca, Fe, and Al, which indicates that further changes to the fuel mixture are necessary. Because of the difference in the chemical association of P in sewage sludge compared to biomass, the ash transformation of P in sewage sludge mixtures differs from biomass for a similar fuel ash composition in terms of K, Ca, Mg, and P.^40^ The content of Al in the fuel mixture is also important as it may lead to the formation of K-Al silicates, which are detrimental to the formation of K-bearing phosphates.^27,28^

Furthermore, the formation of an ash melt may be of high importance for forming K-bearing phosphates.^39^ A high share of K-bearing phosphates was detected in the fixed-bed ashes from the combustion of a sewage sludge and wheat straw mixture but not when the same fuel mixture was combusted in a fluidized bed.^39^ This difference was attributed to the formation of an ash melt with increased interaction during fixed-bed combustion and a more heterogeneous nature of individual bed ash particles during fluidized bed combustion. A more detailed understanding of the significance of molten ash in forming K-bearing phosphates from sewage sludge and biomass mixtures is needed. In addition, the formation of ash melt is a determinant factor for severe ash-related problems such as slagging during the mono-combustion of sewage sludge^41−43^ and K-rich biomass,^32,44,45^ respectively. To assess the viability of a fuel design approach for the recovery of P from sewage sludge, the effects of melt formation for the process stability and for the formation of K-bearing phosphates need to be investigated.

The primary objective of this study was to determine ash transformations during fixed-bed co-combustion of sewage sludge with K-rich agricultural residues, focusing on phosphorus. The ash transformation of P was elucidated on the basis of the fuel speciation as well as the elemental distribution and the crystalline composition in the residual ash fractions formed in the process. Furthermore, the spatial elemental distribution within each ash fraction was determined. The secondary objective was to investigate the effect of co-combustion on the quantitative slag and fine particulate matter formation with relevance to ash-related issues. Phosphorus recovery is discussed on the basis of fuel design regarding the practical aspects of changing the speciation of P in the ashes while simultaneously managing the risk of ash-related issues.

## 2 Materials and Methods

### 2.1 Fuels

Five different pelletized fuels were produced for the experiments and were based on digested sewage sludge (SS), wheat straw (WS), and sunflower husks (SH). These fuels have been used in previous single-pellet macro-TGA studies,^27,28^ and the predicted ash compositions have been evaluated using thermodynamic equilibrium calculations.^29^ The SS used in this study was received as dried and sanitized (120 °C) granules provided by a municipal wastewater treatment facility in the south Stockholm area (Sweden). The process uses iron(II) sulfate to precipitate phosphorus from the wastewater and polyaluminum chloride for subsequent coagulation and flocculation. The WS was provided by Heros Tiernahrung Produktions (GmbH, Thüringen, Germany), whereas the SH was provided by Europack (Bulgaria). The fuels were received as dry 6 mm pellets. Approximately 60 kg of pellets was produced from each fuel mixture. First, the base fuels were crushed in a hammer mill (Bühler, DFZK 1.1 mm sieve) and subsequently homogenized in a ribbon mixer. Fuel blends were mixed on a dry basis (wt % d.b.), and water was added to give a final moisture content of ∼10 wt % before pelletization in a semi-industrial scale pelletizer (SPC 300, 8 mm diameter). The morphology and particle distribution in the pellets were analyzed and discussed previously.^27^

The total ash content and elemental composition of the produced pellets based on three representative fuel samples are given in Table 1. The ashes of WS and SH have high relative concentrations of K and intermediate to low levels of Ca and Mg but differ in the content of Si. By contrast, SS has a high relative concentration of Ca, Fe, Si, and P but low levels of K and Na. Blends of agricultural residues with SS would significantly increase the relative share of K in the blend. However, they differ in the amount of added Si depending on the biomass fuel used. Two blends were made at a higher level of agricultural residue addition, i.e., 90 wt % d.b. wheat straw or 85 wt % d.b. sunflower husks with 10/15 wt % d.b. sewage sludge. Two blends were made at a lower addition, i.e., 70 wt % d.b. wheat straw or 60 wt % d.b. sunflower husks with 30/40 wt % d.b. sewage sludge. The purpose of admixing a low share of SS (WSS10, SHS15) was to add sufficient K, Ca, and Mg to enable the formation of K(Ca, Mg)PO_4_ on a stoichiometric basis. For the mixes with a higher share of SS (WSS30, SHS40), the intention was to attain a relative concentration of K, Ca, and Mg to P such that some amount of Fe or Al would be required stoichiometrically to fill out an orthophosphate structure, i.e., (K, Ca, Mg, Fe, Al)_*x*_(PO_4_)_*y*_.

**Table 1:** CHN Content, Ash Content (AC), and Main Ash-Forming Elements (ICP-AES, IC) of Fuels and Fuel Mixtures ±1 Standard Deviation for the Ash Analysis Based on *n* Replicates^a^

		pure fuels					co-pelletized fuel mixes			
	unit	SS	WS	SH	SW	SS33^b^	SHS40	WSS30	SHS15	WSS10
C	wt % d.b	37.9	47.5	51.3	50.7	46.4^b^	46.2^b^	44.7^b^	49.5^b^	46.6^b^
H		5.2	5.8	6.3	5.1	5.1^b^	5.9^b^	5.6^b^	6.2^b^	5.7^b^
N		5.1	0.4	0.7	<0.1	1.8^b^	2.4^b^	1.8^b^	1.3^b^	0.8^b^
AC		32.8 ± 0.0	4.1 ± 0.0	2.8 ± 0.1	0.3	11.2^b^	14.3 ± 0.1	12.6 ± 0.1	6.8 ± 0.1	6.8 ± 0.3
K	mmol/kg d.b	100 ± 1	210 ± 3	190 ± 3	11	41^b^	158 ± 2	181 ± 7	175 ± 1	196 ± 3
Na		104 ± 2	2 ± 0	<0.5	1	35^b^	40 ± 0	31 ± 1	15 ± 0	12 ± 0
Ca		678 ± 5	69 ± 1	89 ± 3	20	239^b^	322 ± 0	257 ± 9	176 ± 2	127 ± 7
Mg		155 ± 1	29 ± 0	72 ± 2	5	55^b^	105 ± 0	66 ± 2	84 ± 1	41 ± 2
Fe		953 ± 4	2 ± 0	2 ± 0	0	318^b^	376 ± 2	285 ± 10	139 ± 2	93 ± 6
Al		561 ± 2	6 ± 0	1 ± 0	1	188^b^	217 ± 2	169 ± 2	80 ± 2	58 ± 2
Si		1023 ± 5	330 ± 6	11 ± 0	6	345^b^	407 ± 4	548 ± 16	161 ± 7	399 ± 19
P		934 ± 5	19 ± 0	23 ± 1	2	313^b^	383 ± 2	297 ± 10	156 ± 2	107 ± 6
S		378 ± 5	20 ± 0	39 ± 1	2	127^b^	173 ± 2	126 ± 4	89 ± 1	53 ± 3
Cl		27 ± 0	57 ± 0	10 ± 0	2	10^b^	17 ± 0	47 ± 1	12 ± 0	54 ± 0
Zn		8 ± 0	<1 ± 0	<1 ± 0	<1	2.7^b^	3.5 ± 0	3 ± 0	1 ± 0	1 ± 0
Sum		4921	744	437	50	1682^b^	2202	2010	1088	1141
n		3	3	3	1		3	3	3	3

a Adapted with permission from refs (27) and (28). Copyright 2021 and 2022 Creative Commons CC-BY.

b Calculated values based on the fuel mixing ratio.

Because of the poor combustion performance of SS in the system used, it was necessary to lower the ash content by diluting the SS pellets with a supplementary low ash fuel to achieve adequate combustion performance. To this end, the sewage sludge pellets were mixed with softwood (SW) 8 mm pellets (SCA, Luleå, Sweden) at a 1:2 ratio (SS33), which allowed for adequate combustion performance without significantly altering the overall ash composition of the pellet mixture (SS ash >97 wt % of total).

### 2.2 Combustion Experiments

**Figure 1:**
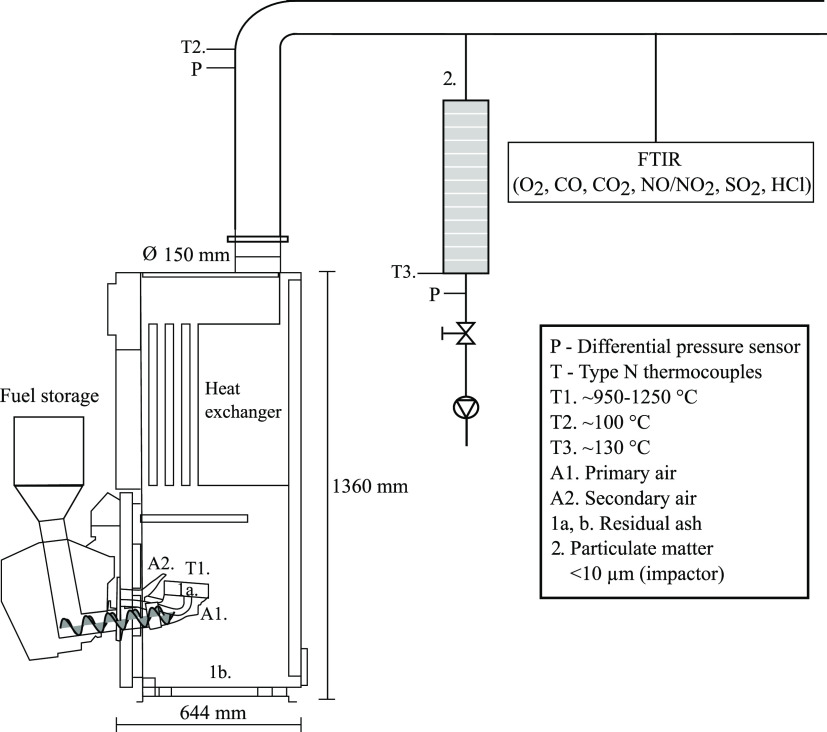
Schematic overview of the underfeed pellet burner (20 kW nominal) in the reference boiler, ash sampling points, flue gas measurements, and temperature and pressure measurements. Adapted from Hedayati et al.^46^ Copyright 2022 American Chemical Society.

The fuels WS and SH and the fuel mixtures SS33, SHS40, WSS30, SHS15, and WSS10 were combusted in an underfed fixed-bed pellet burner (Ecotech Bioline 20) with a nominal output of 20 kW_th_ based on the combustion of wood pellets, installed in a reference boiler (see Figure 1). The boiler is equipped with an integrated heat exchanger and water-jacketed walls. The pellets were fed into the burner cup using a screw conveyor operating at a constant frequency of 25 or 30 Hz (SS33) to achieve a similar thermal output (10.6–14.4 kW) based on the fuel’s bulk density and heating value. Air is supplied through the fuel bed by a frequency-controlled fan (30–40 Hz) that pushes air through vertical slits that are distributed along the inner ring of the burner cup (primary air, point A1) and above the fuel bed through a nozzle (secondary air, point A2). A stoichiometric surplus of O_2_ was already supplied in the primary air inlet. The frequency was manually adjusted to achieve ∼10 vol % O_2_ in the flue gas (λ ∼1.9). For the experiment with SH, the excess O_2_ in the flue gas decreased at constant operational settings, resulting in a median O_2_ value of 6.3 vol %. This was likely caused by an increase in the share of fuel powder in the feeding screw throughout the experiment but had an insignificant impact on the combustion performance. Ash removal from the burner cup is aided by a rotating outer rim that turns clockwise with each revolution of the conveyor screw.

An overview of the operational conditions during each experiment is given in Table 2. Each experiment was initiated with 0.5 kg softwood pellets, except for SS33, which was initiated with 3 kg due to expected slagging issues. The duration of the experiment was adjusted to allow for three particulate matter measurements using a low-pressure impactor at steady-state conditions or approximately 5 ± 1 h. The flue gas concentrations of O_2_, CO, CO_2_, NO/NO_2_, SO_2_, and HCl were continuously measured by Fourier transform infrared spectroscopy (FTIR, Gasmet DX 4000) and logged every 30 s.

**Table 2:** The Experiment Duration; Average Fuel Mass Flow; Average Fuel Load (*P*_th_); and Minimum, Maximum, and Median Concentration of O_2_ and CO in the Flue gas for the Combustion Experiment^a^

fuel	duration (h)	mass flow (kg/h)	*P*_th_ HHV (kW)	O_2_ concentration (mol %) min–max, median	CO concentration (ppm) min-max, median
SH	4.1	2.9	14.4	1.1–16.2, 6.3	3860–8240, 5960
WS^46^	1.8^b^	2.2	11.0	8.4–16.4, 13.5	100–2000, 630
SS33	5.3	2.7	12.8	6.8–15.5, 10.4	40–2330, 150
SHS40	5.8	2.8	12.5	4.6–16.9, 9.8	30–2920, 280
WSS30	6.3	2.4	10.6	7.3–18.7, 12.1	130–3050, 720
SHS15	5.2	2.9	14.4	7.2–13.3, 9.1	60–620, 200
WSS10	5.9	2.7	12.6	6.9–18.6, 10.7	80–4810, 260

a The concentration of CO is normalized to 10% O_2_.

b The duration of the experiment had to be cut short because of severe slagging.

**Figure 2:**
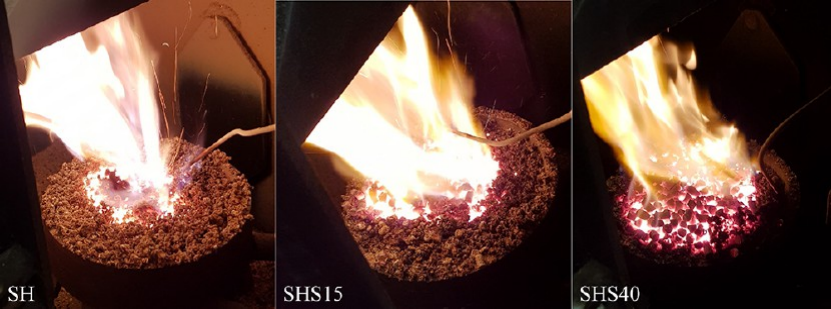
A visual comparison of the combustion zone between the combustion experiments with SH, SHS15, and SHS40.

A shielded N-type thermocouple was used to estimate the peak combustion temperatures during the experiments (see T1 in Figure 1), which, except for SS33 and SH, were measured to be ∼1200 °C. On the basis of a visual inspection of the flame and previous experience with the burner, the peak temperatures measured during the SS33 and SH experiments (∼950 °C) were likely an underestimation of the actual peak temperature. During combustion of a variety of woody and agricultural biomass types using similar operational settings, the temperature in the grate was estimated to be in the range of 1100–1250 °C using three shielded N-type thermocouples.^47^ On the basis of point measurement throughout the fuel bed and the composition and morphology of the residual ash samples, the temperatures in colder regions of the bed are likely to be several hundred degrees lower than the peak combustion temperatures. A visual inspection of the flame indicated significant differences in the temperature distribution across the burner cup and between fuel mixtures (see Figure 2).

The concentration of particulate matter in the flue gas was determined using a preheated (∼130 ± 15 °C) 13-stage low-pressure impactor with nongreased aluminum foil substrates. Nongreased substrates were used to facilitate SEM/EDS and XRD analysis of the PM_1_ ash fraction. Triplicate measurements were taken at the start, middle, and end of the experiment once stable process conditions were established. The experiment with WS had duplicate measurements due to the shorter experiment duration.

After each experiment, residual matter in the burner cup (1a) and bottom of the boiler (1b) was removed after the boiler had cooled down (see Figure 1). Any partially converted fuel pellets or pieces of char larger than 1.4 mm were manually removed from the collected material and subtracted from the mass and energy balance, with the remaining matter denoted as “residual ash”.

An overview of the fractionation, postprocessing, and analytical methods for the collected ash fractions is given in Table 3. The residual ashes were separated into three fractions by sieving (mesh size 1.4 and 3.15 mm), with the fractions denoted as fine bottom ash (<1.4 mm), coarse bottom ash (1.4–3.15 mm), and slag (>3.15 mm). The slag fraction was further fractionated on the basis of size and durability into slag breakable by hand, or “weak slag”, and larger, more durable pieces of slag, denoted as “hard slag”. The concentration of submicron particles (PM_1_) was determined by the amount of material in substrates 1–7 (*D*_50%_ 0.022–0.613 μm), and the concentration of particles in the 1–10 μm size range (PM_1–10_) was determined by the amount of material in substrates 8–13 (*D*_50%_ 0.961–10.249 μm). *D*_50%_, or the “cutoff size”, corresponds to the aerodynamic diameter of particles trapped with an efficiency of 50% given a particle density of 1 g/cm^3^.

**Table 3:** Overview of the Fractionation and Postprocessing of the Collected Ash Fractions

collected ash	particle size (sieving)	ash fraction	postprocessing	analysis method
residual ash sampling points 1a and 1b in Figure 1	>3.15 mm	hard slag^a^	milling <1.4 mm, milling 5–50 μm, epoxy resin^b^	SEM/EDS (cross section^b^), XRD (5–50 μm), elemental analyzer (5–50 μm)
		weak slag^a^	milling <1.4 mm, milling 5–50 μm	SEM/EDS (<1.4 mm), XRD (5–50 μm), elemental analyzer (5–50 μm)
	1.4–3.15 mm	coarse bottom ash	milling <1.4 mm, milling 5–50 μm	SEM/EDS (<1.4 mm), XRD (5–50 μm), elemental analyzer (5–50 μm)
	<1.4 mm	fine bottom ash	milling 5–50 μm	SEM/EDS (<1.4 mm), XRD (5–50 μm), elemental analyzer (5–50 μm)
low-pressure impactor (13 stages) sampling point 2 in Figure 1	*D*_50%_ 0.961–10.249 μm	substrates 8–13	too little sample quantity	
	*D*_50%_ 0.022–0.613 μm	substrates 1–8		SEM/EDS (substrates 3, 4, 5, 6), XRD (substrates 3, 4, 5, 6)

a Hard and weak slag fractions were fractionated by hand on the basis of size and durability

b Hard slag pieces were cast in epoxy resin, cut, and dry polished with SiC grinding paper.

### 2.3 Chemical Characterization

To enable SEM/EDS analysis, subsamples of coarse bottom ash, weak slag, and hard slag were produced by manually crushing the samples to a particle size where they would pass a sieve with an aperture size of 1.4 mm. Subsamples of the crushed residual ash fractions and fine bottom ash were further milled to ∼5 μm using a ball mill (Retch MM 400, Ø 5 mm tungsten carbide balls) for XRD and elemental analysis. Representative subsamples of the residual ash fractions and crushed residual ash fractions were collected using a rotary sample splitter (Retsch PT 100).

Morphology, elemental composition, and spatial distribution were studied using SEM (Jeol JSM-IT300) equipped with a backscattered electron (BSE) detector and an Oxford EDS detector. Before analysis, the fine or crushed samples (PM_1_, fine BA, coarse BA, weak slag) were mounted on carbon tape and attached to aluminum sample holders. For the PM_1_ fraction, the most particle-laden substrates, usually 3, 4, and 5, were used for the bulk elemental analysis, which constituted >80 wt % of the total PM_1_ fraction. The exception was the SH experiment, which had the most material in substrates 4, 5, and 6. The PM_1–10_ fraction was not analyzed as the sample amount was too low (<100 μg). In addition, several representative slag pieces from the “hard slag” fraction of each experiment were cast in epoxy resin (Struers EpoFix), cut, and dry polished with SiC grinding paper to investigate the interior of the slag.

For the bulk elemental composition, an average was calculated from at least three mappings (analysis area: 1.2 × 1.6 mm) distributed throughout the ash sample. In all mappings, an analytic resolution of 1024 × 768 was used, with each pixel representing one point analysis. The bulk analysis was complemented by the characterization of distinctive regions and particles in the sample using the same resolution, i.e., the spatial distribution of elements. The area analysis was performed by mappings at higher magnification (analysis area: 0.512 × 0.384 mm). On the basis of the BSE image and mapping data, characteristic particles and regions in the sample were identified, and the elemental compositions of the area were obtained from the mapping data. By combining three to seven area analyses of each characteristic particle or region in the sample, an average elemental composition was estimated for ash-forming elements with a concentration of >1 mol % on a C- and O-free basis. In addition, statistical analysis was used to compliment the compositional trends observed in the area analysis of characteristic particles and regions. For the statistical analysis, more than 230 randomly distributed point analyses were used to determine percentile distributions and trends in composition between the detected elements, e.g., positive and negative spatial correlations between the main ash-forming elements.

The milled ash fractions were analyzed by powder X-ray diffraction (XRD, Panalytical Empyrean, UK), for which they were mounted in a low-background Si sample holder. The analysis was performed with a Cu Kα X-ray tube and an array detector (Pixel3D) with a data collection 2θ range between 10 and 70° and a scan interval of 0.007°. A dual scan was used to reduce the effect of random noise effects. The crystalline phases were identified and quantified using the Rietveld method with the HighScore Plus 4.8 software^48^ and the PDF-4+ 2021 database.^49^ The amorphous share of the sample was quantified using the K-factor method.^50,51^ The samples were analyzed in series with a fully crystalline Si reference sample in the middle of the series to establish the instrument intensity constant, the so-called K-factor. The K-factor enabled the quantification of amorphous material in the sample by setting the Rietveld quantification on an absolute scale.

The amounts of residual C, H, and N in the finely milled bottom ash and slag fractions (5–50 μm) from the co-combustion experiments were determined using an elemental analyzer (Euro EA3000, standard: EN 15104).

## 3 Results and Discussion

### 3.1 Mass Distribution of Ashes and Acidic Emissions

**Figure 3:**
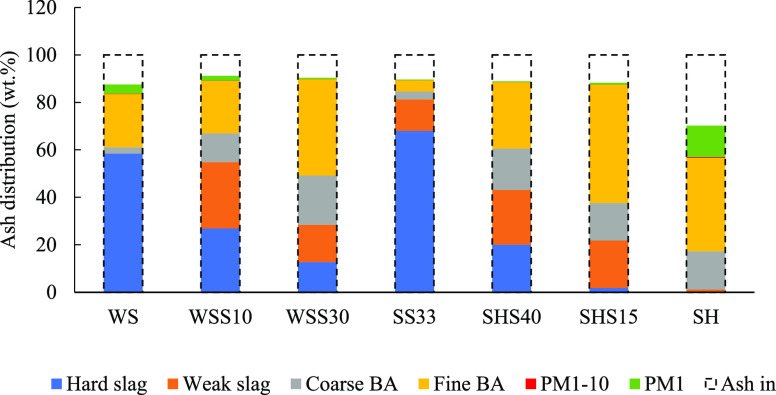
Distribution of ash (wt %) between hard slag, weak slag, coarse BA, fine BA, PM_1–10_, and PM_1_ on a CHN-free basis. The collected ash fractions are normalized to the theoretical amount of ash fed with the fuel based on a standard ashing test (550 °C).

On the basis of the ashes collected in the reactor (slag, bottom ash) and in the impactor measurements (PM_1_, PM_1–10_), a mass balance was established, and the mass closure was determined. The distribution of ashes between the collected ash fractions normalized to the theoretical amount of ash fed with the fuel is shown in Figure 3. In all cases, most of the collected ash was found in the bottom ash and slag ash fractions. The mass closure was similar for fuel mixtures containing SS (88–92 wt %). The mass closure of SH was lower than for the other fuel mixtures (82 wt %) and produced a significantly higher share of PM_1_ (13 wt %). The amount of material on the boiler walls and heat exchanger increased with the general release of particulate matter and is likely one of the reasons for the lower mass closure of SH compared to the other fuel mixtures. Deposition of SH ash on boiler interiors amounted to about 10 wt % of the ingoing ash, whereas deposition for the other fuel mixtures was below 2 wt % of the ingoing ash. The low amount of PM_1–10_ across all experiments is due to the boiler design, which captures particles of this size range inside the boiler.

The lower yields of collected ash could be explained by an overestimation of the theoretical input of ash, especially for fuels that may form a high share of carbonates, sulfates, and chlorides such as SH. The low temperature of a standard ashing test (550 °C) would stabilize higher quantities of carbonates, sulfates, and chlorides in the condensed phase compared to the experiments, possibly causing a lower mass closure due to the higher volatilization of elements from carbonates, sulfates, and chlorides above 1100 °C. The ash yield from the WS experiment was slightly above 100%, which was attributed to char remaining in the ashes.^46^ In addition, significant concentrations of HCl (median 14–58 ppm) and SO_2_ (median 109–345 ppm) were measured in the flue gas during the experiments with sewage sludge mixtures, which are not accounted for in the mass balance. The concentration of acidic gases in the SH and WS experiments was low, with a median value of 1 or 11 ppm of SO_2_, respectively, and 0.2 ppm of HCl in both experiments.

The amount of residual CHN in the slag and bottom ash fractions from the co-combustion experiments was similar (3.1–4.5 wt %) and primarily consisted of carbon with a median C-share of 95 wt %. The average CHN residue in the residual ashes of the mono-combustion experiments of SH and WS was significantly higher at about 6 and 16 wt %, respectively. The CHN share was significantly lower in the slag fractions compared to the BA fractions.

Less than 1 wt % slag was found in the residual ashes from the combustion of SH, whereas the combustion of SS and WS^46^ resulted in more than 60 wt % slag. The SHS mixtures resulted in a significant reduction in the slag amount compared to SS, whereas the WSS mixtures resulted in less slag than both the original fuels.

**Figure 4:**
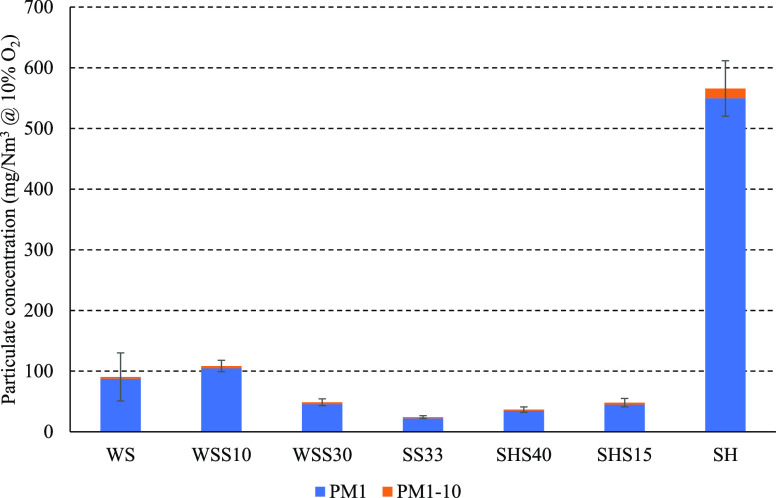
The concentration of PM_1_ and PM_1–10_ in the flue gas with error bars indicating ±1 cumulative standard deviation (WS: *n* = 2, other fuels: *n* = 3).

The particle concentration in the flue gas was determined on the basis of the mass collected in the impactor measurements during the process. Figure 4 shows the normalized concentration of PM_1_ and PM_1–10_ in the flue gas. The trends show a decrease in particulate matter formation when increasing the share of SS in the fuel mixture, with the minimum value obtained during SS33 combustion.

### 3.2 Elemental Concentration and Distribution in PM_1_, Bottom Ash, and Slag

**Figure 5:**
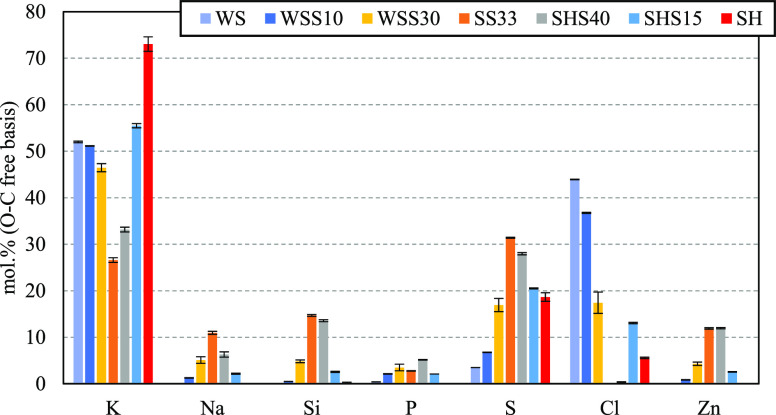
Average elemental composition on an O- and C-free basis (mol %) of PM_1_ (<1 μm) based on three SEM/EDS mappings. Error bars indicate ±1 standard deviation.

A visual comparison of the impactor stages 1–7 indicated a low fraction of soot in the PM_1_, with the deposited particles having a white or light gray color (SH, WS, SHS40, SHS15, WSS10) or a gray or dark gray color (SS, WSS30). The PM_1_ was dominated by K and varying amounts of S and Cl (see Figure 5). The quantities of Mg, Al, Ca, and Fe were below 1 mol % in all the PM_1_ samples. On the basis of the SEM/EDS mapping, Si was present in very fine particles evenly distributed throughout the sample. The concentration of P in PM_1_ was very low in all experiments.

The PM_1_ from the WS experiment was dominated by K and Cl with lower amounts of S.^46^ The elemental composition matched the identified crystalline phases in the PM_1_ sample, which comprised KCl and minor amounts of K_2_SO_4_.

**Figure 6:**
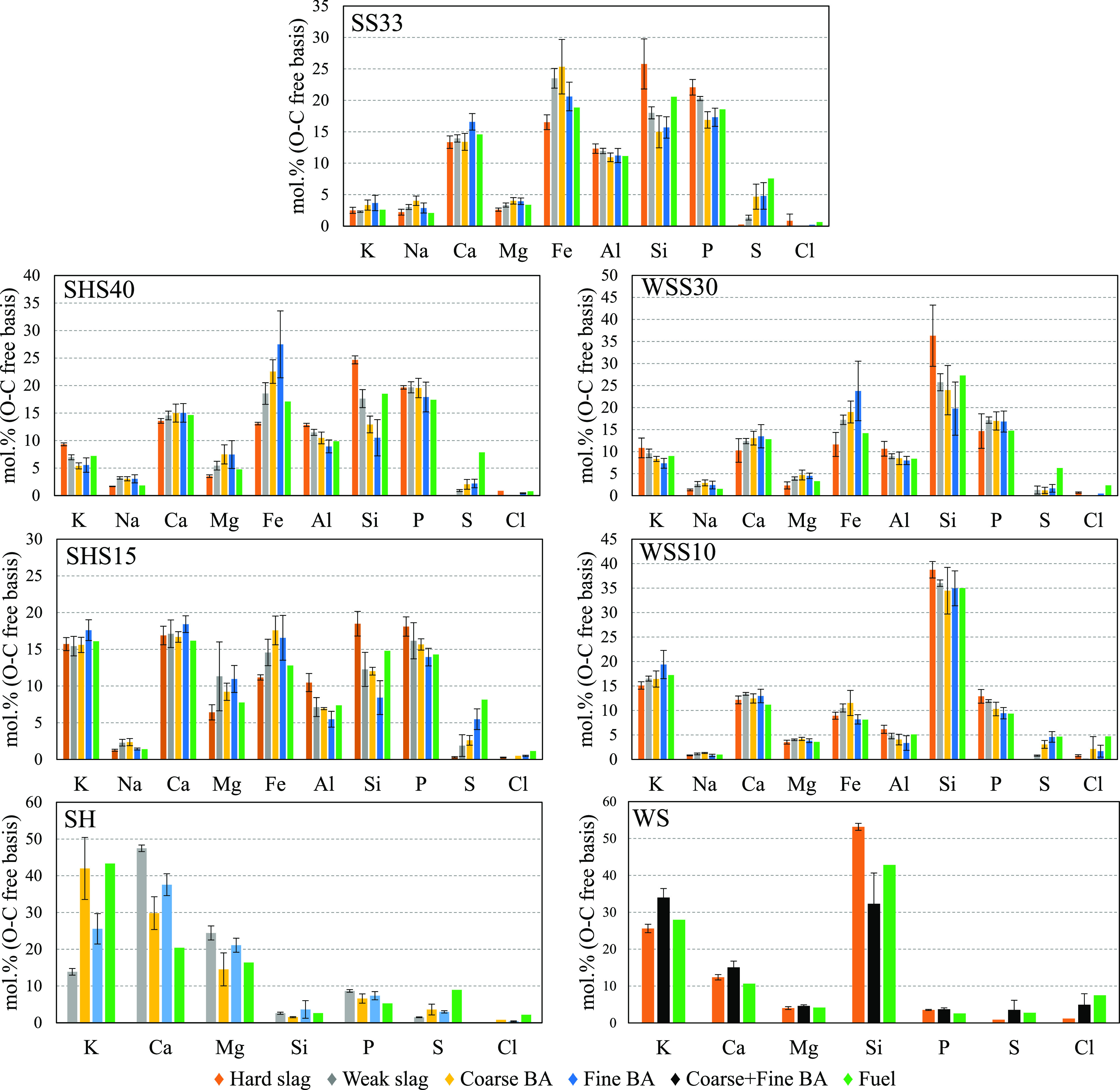
Average elemental composition (mol %, O- and C-free basis) of hard slag, weak slag, coarse bottom ash, fine bottom ash, and fuel composition based on three to seven SEM/EDS mappings. Error bars indicate ±1 standard deviation.

Compared to the fuel ash composition, the bottom ash and slag fractions were depleted in sulfur and chlorine, with the concentration decreasing with particle size and sintering degree (see Figure 6). Si showed enrichment in the slag fractions and depletion in the BA fractions compared to the initial fuel compositions. The opposite trend was observed for Fe. The residual ash fractions of SH were enriched in Ca and Mg but depleted in K compared to the original fuel. Na, Al, and Fe are not shown for the ashes of WS and SH because of low concentrations (<1 mol %).

Overall, the mass closure for elements K, Ca, Mg, Fe, Al, Si, and P of the SS mixtures was within expectation, with a median value of 100 ± 16 wt %. Outliers and larger ranges of uncertainty were found for elements that represented very low concentrations in the original fuels and the collected ashes, e.g., Na. The distribution of elements between the different ash fractions was similar for P, Ca, Fe, Al, and Mg in all the co-combustion fuel ashes. The mass closure for Cl and S was low (<50 wt %) for the SS mixtures, but this was expected given the concentration of acidic gases in the flue gas.

**Figure 7:**
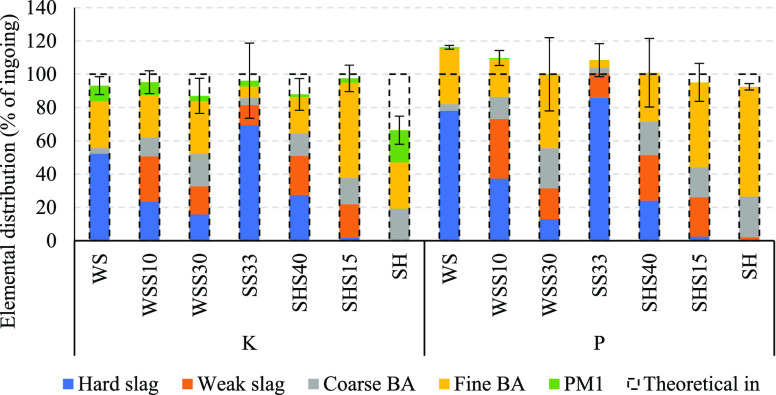
Elemental distribution of K and P between the collected ash fractions normalized to the theoretical amount of K and P fed with the fuel mixture. The standard deviation is the cumulative uncertainty of the elemental composition based on the standard deviation of each ash fraction (*n* = 3–7).

The elemental distribution of K and P between the collected ash fractions normalized to the amount of ingoing K and P is shown in Figure 7. The majority of K and P was captured in the residual ash fractions, i.e., slag and bottom ash, with an overall mass closure of 84–95 wt % for K and 95–109 wt % for P. The mass balance of elements for SH and WS^46^ showed a lower mass closure for K with a higher share in the PM_1_ fraction.

On the basis of the formation of PM_1_ and the share of slag, the co-combustion significantly improved the combustion performance of SS, SH, and WS regarding the release of potentially deposit-forming K and slag formation. The high retention of K in bottom ash and slag and the presence of acidic gases in the flue gas from the co-combustion experiments indicate that the release of K was the main limiting factor for the formation of PM_1_.

However, the increased retention of K in bottom ash and slag did not cause an increase in the share of slag that formed. The combustion experiments with SS33 indicated a severe risk for slag-related operational issues due to the buildup of resilient ash deposits on the burner grate. SHS mixtures were the most effective at reducing the slagging tendency of SS, with the amount and durability of the slag decreasing with the share of SH. The experiment with pure WS suggested a significant risk of slag-related operational issues, and the experiment had to be cut short because of slag buildup on the burner cup.^46^ The WSS mixtures produced lower shares of slag, and the slag durability was decreased when compared to the original fuels SS and WS. The WSS10 mixture had a larger slagging tendency than the WSS30 mixture, which shows that increasing the share of WS would increase the amount of slag formed even further. The slag share and durability are consistent with a previous study investigating the same fuel pellets in a macro-TGA at 800 and 950 °C.^27^

The small difference in bulk composition between the different residual ash fractions implies that ash fractionation and melting behavior are likely not the sole explanation for the difference in particle size and sintering degree between these ash fractions. It would likely be necessary to consider the combined effects of temperature distribution and melting characteristics of the ashes to estimate the amount of slag that forms for a given fuel.

### 3.3 Morphology and Spatial, Elemental, and Phase Distribution in the Bottom Ash and Slag

**Figure 8:**
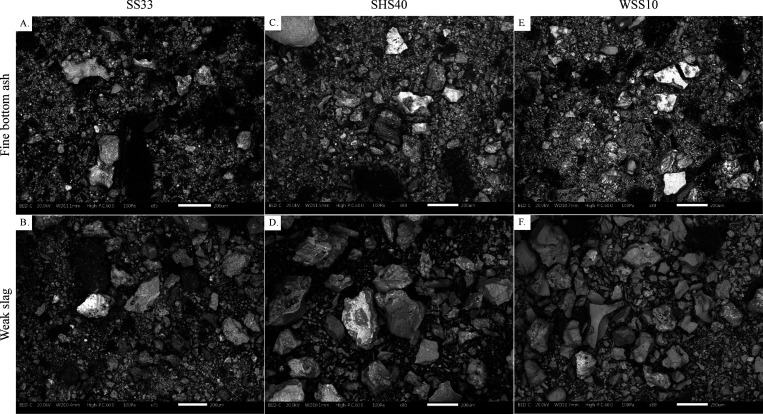
SEM-BSE micrographs of ash fractions: (A) SS33 fine BA, (B) SS33 weak slag, (C) SHS40 fine BA, (D) SHS40 weak slag, (E) WSS10 fine BA, and (F) WSS weak slag at 75–85× magnification.

**Figure 9:**
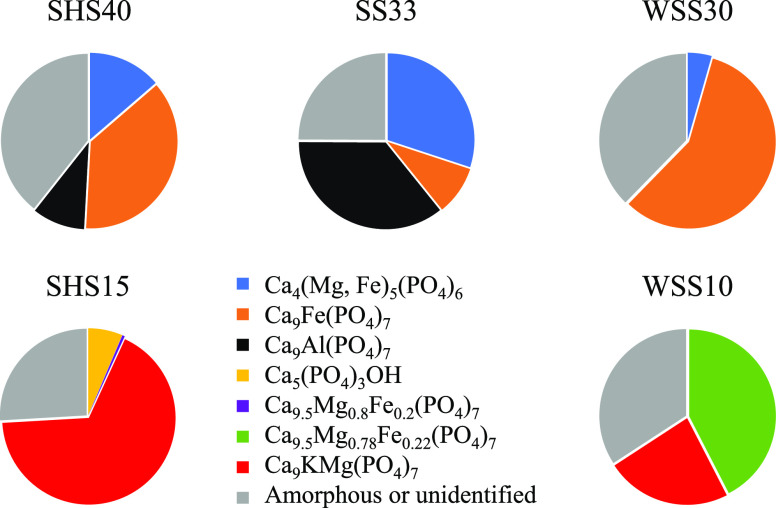
Mass distribution of P between crystalline and amorphous phases in the residual ash based on the mass balance of the bottom ash slag ash fractions, the bulk elemental composition, and the quantitative XRD results.

**Figure 10:**
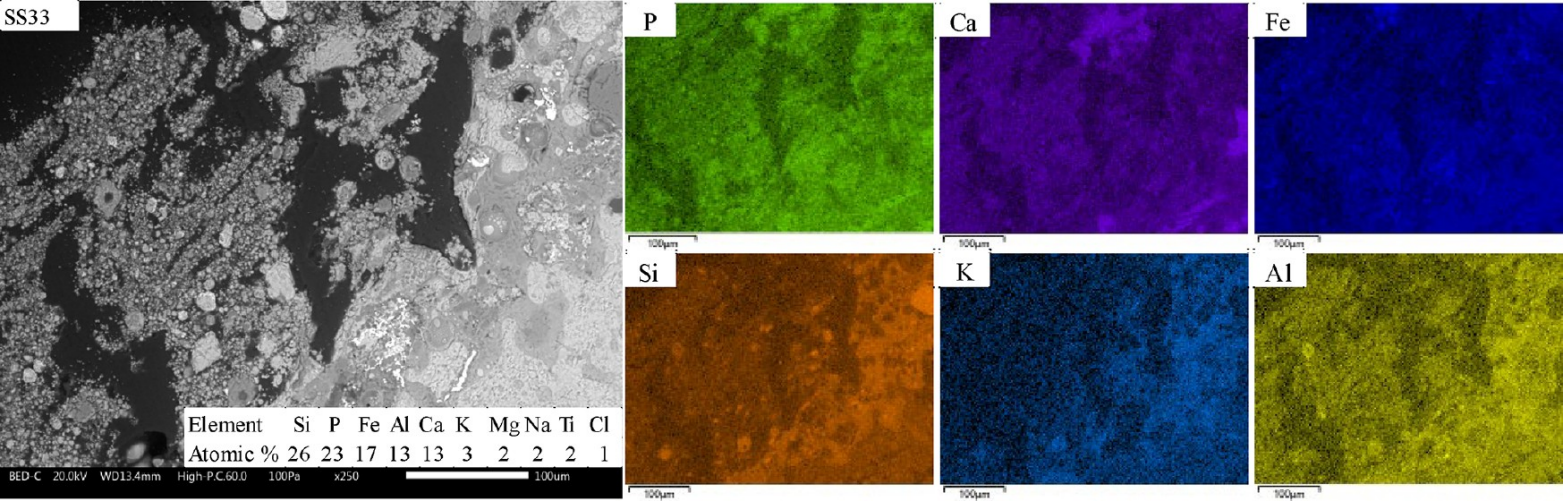
BSE micrographs and SEM/EDS mappings within the image area of hard slag cross sections from SS33 at ×250 magnification.

**Figure 11:**
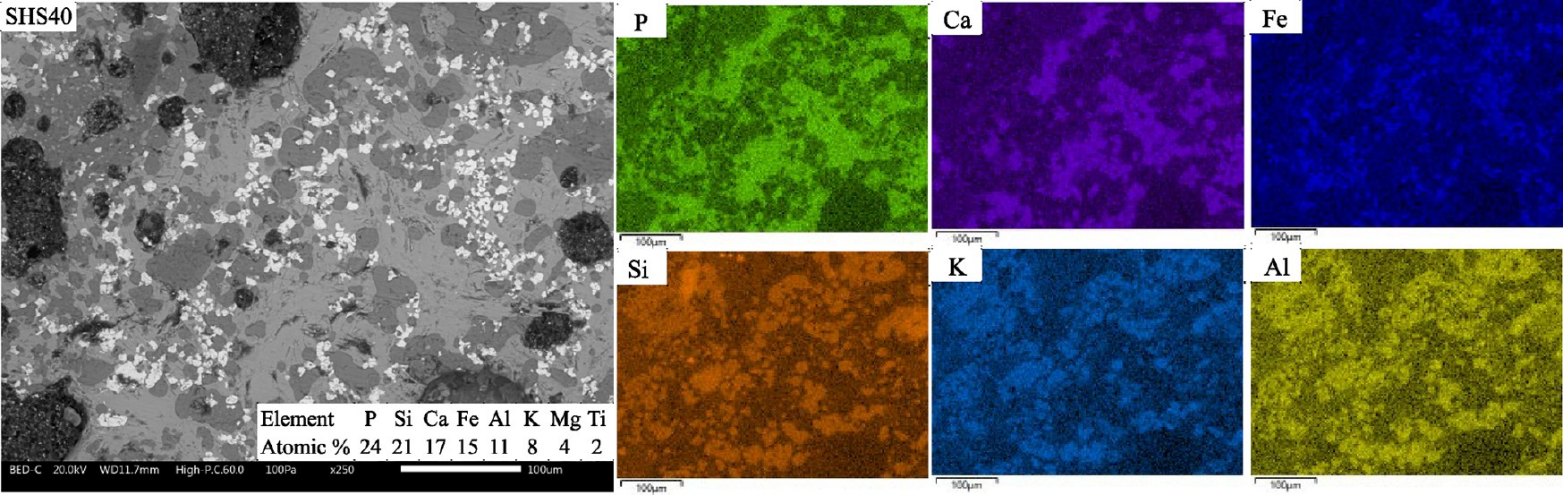
BSE micrographs and SEM/EDS mappings within the image area of hard slag cross sections from SHS40 at ×250 magnification.

**Figure 12:**
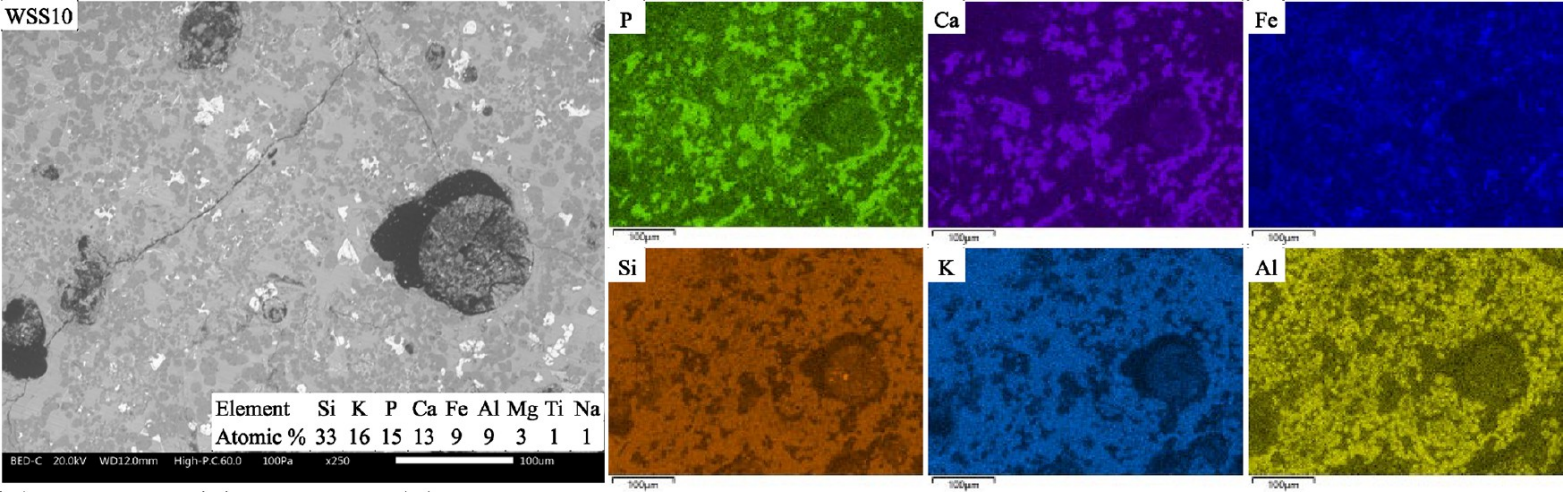
BSE micrographs and SEM/EDS mappings within the image area of hard slag cross sections from WSS10 at ×250 magnification.

Within each fuel mixture, the residual ash fractions were quite similar concerning the morphology (Figure 8), spatial distribution of elements (Figures 10–12), identified crystalline phases, and amorphous share (Table 4). Only small differences could be observed in the fate of P for the residual ash between the SHS40 and WSS30 mixtures or the SHS15 and WSS10 mixtures (see Figure 9).

**Table 4:** The Identified Crystalline Phases (XRD) of All the Residual Ash Fractions^a^

	SS33				SHS40				WSS30				SHS15				WSS10				WS		SH		
chemical formula	1^b^	2^b^	3^b^	4^b^	1	2	3	4	1	2	3	4	1	2	3	4	1	2	3	4	1	4	1	2	3
Ca_4_(Mg, Fe)_5_(PO_4_)_6_	12	15	12	17		7	10	11		2	3	6													
Ca_9_Fe(PO_4_)_7_	16	19	21		23	22	20		23	24	21	20													
Ca_9_Al(PO_4_)_7_				23				19																	
Ca_9_KMg(PO_4_)_7_													22	28	31	20	17	19							
Ca_9.5_Mg_0.8_Fe_0.2_(PO_4_)_7_																8									
Ca_9.5_Mg_0.78_Fe_0.22_(PO_4_)_7_																			21	19					
Ca_5_(PO_4_)_3_OH													5								4		17	15	16
CaKPO_4_																					1	2	7	8	8
KAlSiO_4_														15											
K_0.96_AlSiO_4_															18	9									
KAlSi_2_O_6_					13	15	14	15	15	14	14	18	11	13	12	11	10	16	16	13					
Ca(Mg, Fe, Al)Si_2_O_6_																						4			
SiO_2_ (quartz)	1	1	2	1	8	5	3	0	1	4	3	2	2	1	0	1		2	0	5	1	1			
SiO_2_ (cristobalite)																	4								
Fe_2_O_3_	11	14	15	17	16	17	15	10	14	15	13	7	7	2	1	6	5	4	3	1					
Fe_3_O_4_												2	7	10	12	9									
CaSO_4_	3																								
CaCO_3_												0									3		3		3
(Ca,K_2_)CO_4_																							9	5	4
Ca(OH)_2_																							5	10	9
K_2_SO_4_													7		1		1				5		10	9	7
Ca_2_K_2_(SO_4_)_3_																					2				
MgO																							15	18	18
KCl																	1				5				
amorphous	58	50	51	42	41	34	37	44	47	42	45	45	39	31	25	36	63	58	60	63	79	93	35	35	36

a The quantification (wt %) was performed using Rietveld analysis with the amorphous share of the sample estimated using the K-factor method. WS data were from Hedayati et al.^46^

b (1) Fine bottom ash, (2) coarse bottom ash, (3) weak slag, and (4) hard slag.

Figure 8 shows an overview of the morphology of a selection of fine BA fractions and weak slag fractions. The fine BA fraction and the weak slag fraction were chosen for the comparison as they showcase the largest differences in morphology among the fine BA, coarse BA, weak slag, and hard slag fractions. The images of WS30 and SHS15 are not shown for brevity as they are similar to the BSE of SHS40 and WSS10, respectively. The crushed weak slag fraction was used to enable a visual comparison at a similar resolution to the fine BA fraction. All residual ash fractions contained a fraction of ash particles with a morphology that suggested that it had been previously molten, i.e., mostly solid particles with a smoother outer surface and a more uneven, jagged interior in the case of the crushed slag. Additionally, the BA fraction of SS33 and, to a lesser extent, the BA of the SHS and WSS mixtures contained large, sintered particles (>500 μm) with a very porous structure resembling pure SS ash. Occasionally, the surface of the sintered particles was covered in a layer of molten ash or by globules of molten particles approximately 5–30 μm in diameter. No ash particles with a composition close to the composition of SH or WS were detected in the slag or bottom ash fractions, suggesting thorough interaction of the agricultural ash with the SS ash.

On the basis of SEM-BSE images of the particles in the residual ash fractions, visually similar characteristic particles were present in all samples. Although the bulk composition of these regions varied with the fuel ash composition, the enrichment of elements followed a similar pattern for all mixtures with SS. The brightest particles or regions are enriched in Fe, which are spatially accumulated near the particle surface or small grains distributed throughout the inside of the slag particles. The color gradient from light gray to dark gray generally corresponds to regions with high or low concentrations of cations, with P being enriched in the cation-rich regions. In contrast, Si and Al are enriched in the cation-poor regions. Smaller amounts of extraneous silicate minerals are also present in the BA.

The XRD results of the fine BA, coarse BA, and weak slag samples are shown in Table 4. Relatively small differences can be observed in the identification and quantification of crystalline phases between the different residual ash fractions of each fuel mixture. In the larger and more sintered hard slag fraction, Ca_9_Al(PO_4_)_7_ and Ca_9.5_Mg_0.8_Fe_0.2_(PO_4_)_7_ were identified, and the share of Ca_4_(Mg, Fe)_5_(PO_4_)_6_ was higher. The XRD patterns also show high quantities of K-Al silicates in the ash fractions from co-combustion, indicating high interaction of Al from SS-ash and K from WS-/SH-ash. Pure Fe oxide was identified in positive correlation to the share of SS-ash in the fuel. Small amounts of sulfates and chlorides could also be identified in the fine BA fractions.

The approximate distribution of elements between the crystalline and the amorphous fractions was calculated for all collected residual ash fractions based on the XRD analysis and SEM/EDS mappings. Crystalline and amorphous distribution data were evaluated for all main ash-forming elements in all the collected ash fractions. In general, P, Ca, and Fe were more crystalline than the bulk of the residual ash, whereas K, Al, Mg, and Si were less crystalline. The P distribution in the bulk residual ashes of the co-combustion experiments is shown in Figure 9. The distribution shows that the P association transforms gradually from SS-dominated ashes toward higher shares of agricultural residues, with Fe and Al in the phosphates being substituted by Ca, Mg, and K. This trend was obtained for all individual residual ash fractions.

A visual inspection of slag cross sections indicates more distinct differences between the samples from the SEM-BSE and EDS analysis (Figures 10–12). However, the color gradients of the slag cross sections shown in Figures 10 and 11 follow the same enrichment patterns as the crushed ash particles in terms of the enrichment of elements.

The SEM/EDS mapping of the SS33 cross section indicates that P is present throughout the entire slag but slightly enriched in the cation-rich regions together with Ca (Figure 10). This observation is supported by the statistical analysis, which shows a positive correlation between the concentration of P and Ca and a negative correlation to Si. XRD analysis identified Ca_4_(Mg, Fe)_5_(PO_4_)_6_, Ca_10_Al(PO_4_)_7_, and Fe_2_O_3_ as the main crystalline phases in the hard slag (see Table 4). A small amount of SiO_2_ was detected, which likely originated from included minerals.

The mass distribution of crystalline elements indicated that the share of crystalline P (75%), Ca (125%), and Fe (107%) is higher than the bulk amorphous share of the sample (42%). In contrast, K, Si, Al, and Mg are almost exclusively in the amorphous fraction; i.e., <10% of the element was detected in the crystalline phases. Furthermore, because of the overdetermination of Ca and Fe in the crystalline phases, the amorphous P is unlikely to be associated with Ca or Fe. The SEM/EDS area analysis supports this observation as the bulk composition and spatial distribution of elements of the slag cross section indicate a ratio of Ca to P that is too low (<1) for all P to be present as the identified crystalline mix of Ca_4_(Mg, Fe)_5_(PO_4_)_6_ and Ca_9_Al(PO_4_)_7_.

Figure 11 shows the BSE images of the SHS40 slag cross section and the mappings of elements P, Ca, Fe, Si, K, and Al. Compared to the slag from the SS33, the elements are more clearly separated into three visually distinctive regions. However, the enrichment of elements between the regions is stronger for SHS40 than for SHS15. The statistical analysis of the SHS40 and SHS15 slags showed a positive correlation between P and Ca and a negative correlation with K, Al, and Si. However, the correlation was weaker for SHS15, supporting the general observation that Si and P are more clearly separated in the SHS40 slag than in the SHS15 slag.

In the SHS40 residual ashes, the same phosphate phases were identified as in the SS33 residual ashes but with a lower relative share of Ca_4_(Mg, Fe)_5_(PO_4_)_6_ (Table 4). In the SHS15 sample, Ca_9_KMg(PO_4_)_7_ and Ca_9.5_Mg_0.8_Fe_0.2_(PO_4_)_7_ were identified but not Ca_4_(Mg, Fe)_5_(PO_4_)_6_. Small amounts of SiO_2_ were identified in all three slags, but the SHS slags also identified substantial amounts of KAl(SiO_3_)_2_ and, in the case of SHS15, K_0.96_AlSiO_4_. A high share of crystalline Fe was also identified in all three slags, including a slightly reduced Fe_3_O_4_ phase (SHS15).

The mass balance of crystalline phases indicated a slight decrease in the crystallinity of P (75% → 60, 65%) and Fe (110% → 85, 105%) from SS33 to SHS40 and SHS15, respectively. On the basis of the bulk composition and spatial distribution of elements in the SHS40 and SHS15 slag cross sections, the ratio of Ca to P is too low (>1.06, >1.31) for all P to be present in a stoichiometric mix of whitlockite and stanfieldite phases.

The residual ash from the WSS30 mixture had a similar outcome as the SHS40 mixture, with no significant differences concerning the spatial distribution of elements, the identified phases, and the elemental distribution between the crystalline and amorphous share of the sample. In the WSS10 slag cross sections, the bright, Fe-enriched regions in the SEM-BSE images are less frequent throughout the sample and almost completely absent across large regions (Figure 12). No significant difference could be seen in the statistical analysis between the SHS15 and WSS10 samples, which indicated that P had a negative correlation to K, Al, and Si and a positive correlation to Ca. However, the partitioning of P and Si between the cation-rich and cation-poor regions is more pronounced in the slag of WSS10 than in the slag of SHS15.

The XRD results of the WSS10 slag identified the phases Ca_9.5_Mg_0.8_Fe_0.2_(PO_4_)_7_, KAl(SiO_3_)_2_, SiO_2_ and a small share of Fe_2_O_3_, with the majority of the sample being amorphous. The mass balance of crystalline material indicated that P (55%), Al (55%), and Ca (80%) were more crystalline than the bulk of the sample, whereas Fe (10%), K (25%), Si (30%), and Mg (25%) were less crystalline. Compared to the SHS15 slag, the crystallinity of Fe and Si in the WSS10 slag is significantly lower. As the main difference between the two reference fuels is the amount of reactive Si in the ash mixture, this implies that the Si in WS may inhibit the crystallization of Fe from the slag.

The ratio of Ca to P in the amorphous and unidentified phases of all the residual ashes is too low for all P to form whitlockites, which would indicate that the remaining amorphous P is of a different chemical association. In the ashes from the SHS40 and WSS30 mixtures, the SEM/EDS area analysis indicates that K is commonly present in a ∼1:1 ratio with Al. However, in the SHS15 and WSS10 mixture, the ratio of K to Al is substantially higher than 1, indicating that K-containing phases other than K-Al silicates are likely to have formed.

### 3.4 General Behavior of Phosphorus

For all fuels and fuel mixtures, the share of P in the residual ash fraction was high, with minor shares of P in the PM_1_ fraction. These results are consistent with other lab-scale fixed-bed combustion experiments using biomass and biomass sludge mixtures.^39,46^ However, combustion experiments in an 8 MW grate-fired boiler with biomass and sludge mixtures indicated a depletion of P in the bottom ash fraction and enrichment of P in the filter ash together with Ca, K, Mg, Fe, Pb, and Zn.^26^ Although no enrichment of P was observed in more easily entrained fine or coarse BA fractions for any of the fuel mixtures of this study, the effect of higher flue gas velocities is technology-specific and could cause a larger entrainment of ash than observed here. Depending on the specific combustion technology, P could be entrained in an industrial-scale setting and become part of the cyclone, bag, or electrical filter ashes (PM >1 μm).

For all SS mixtures, across all the ash fractions, the dominant crystalline phosphates identified were whitlockite-type phases with a general structure of Ca_9_K_*x*_(Ca, Mg, Fe)_*y*_(Fe, Al)_*z*_(PO_4_)_7_. Stanfieldite (Ca_4_(Mg, Fe)_5_(PO_4_)_6_) was also a major phosphate phase in the ashes of SS33, SHS40, and WSS30 but decreased in share as more biomass was added and was not identified in the SHS15 and WSS10 ashes.

The share of biomass in the mixture, i.e., the ratio of P to K, Ca, and Mg, had a significant impact on the fate of P, but no obvious difference could be seen between mixtures with SH or WS. In the residual ash from SS33, P was associated with Ca, Fe, and Al, whereas Si was not associated stoichiometrically with any other ash-forming element. For the low-biomass mixtures (SHS40, WSS30), the association of P shifted toward more Ca and Mg at the expense of Fe. At the same time, Si was associated with Al and K in these ashes. This implies that when the availability of K is limited, it is preferably incorporated into K-Al silicates rather than K-(Ca, Mg) phosphates for SS mixtures. Similar outcomes have been observed from the thermochemical treatment of SS ashes using alkali additives, which saw little effect on the solubility of P in alkaline ammonium citrate before a Na/P molar ratio of >1 is reached in the ash.^21^

Melt formation in all the experiments could be ascribed to the melting behavior of the individual fuel ashes. Excessive slagging during WS combustion due to K silicate formation and partial melt formation by carbonate melting during the combustion of Ca-K-rich fuels (SH) were described and discussed previously.^28,46,52^ For SS, the initiation of slag could be correlated to a sintering effect of Fe-rich regions on the particle surface.^27,29^ However, the chemical composition in the slag fractions showed a high degree of incorporation of ash-forming elements from both individual fuels in the respective mixtures. Furthermore, the distinctive regions in the slag cross sections indicate an immiscibility gap in the ash mixture, with a clear partitioning of Si and P between the two melts. Previous studies indicate that high shares of Ca in the ash would be incorporated in a P-rich slag and promote the formation of a secondary Si-rich slag.^52^

As the precipitation of crystalline compounds depends on the composition of each melt phase, the immiscibility gap would affect the type of phosphates that could precipitate. At the higher addition level of SHS15, the immiscibility gap is still present, but the partitioning of Si and P between the two melts is less pronounced. The partitioning of Si and P in the WSS10 slag was higher than in the SHS15 slag but lower than in the slag of the low-biomass mixtures. However, the precipitated phosphate phases in WSS10 and SHS15 remained as whitlockite-type phosphates, which indicate that the change in composition was insufficient to favor the formation of phosphates such as KCaPO_4_ or KMgPO_4_. A similar partitioning of Si and P was observed when P was added to a range of bulk liquid compositions in the two liquid boundaries of the K_2_O-FeO-Al_2_O_3_-SiO_2_ system at 1180 °C.^53^

The lower degree of the partitioning between Si and P in the SHS15 slag compared to the WSS10 slag may be a direct result of the increase in SiO_2_ content. A study investigating the solubility of Ca_5_(PO_4_)_3_OH in cation-lean, Si-rich melts at 1100–1500 °C and 8 kbar pressure showed low solubility and diffusion rates of P, which decreased further with the share of SiO_2._^54^ The observed difference in partitioning is likely caused by the decrease in solubility of Ca-rich phosphate phases in the more Si-rich WSS10 slag.

On the basis of the fate of P (Figure 9), the bulk composition (Figure 6), and the spatial distribution of elements (Figures 10–12), the amorphous or unidentified P is associated with Ca to a lesser degree than the identified crystalline phases. A feasible explanation is that a substantial share of amorphous or unidentified P is associated with Al, Mg, and possibly Fe in the SS33, SHS40, and WSS30 ashes. For the ashes of the SHS15 and WSS10 mixtures, there is excess alkali beyond what is necessary to compensate for the charge of Al^3+^ in a feldspar or feldspathoid structure ((K, Na)AlSi_*x*_O_*y*_). Therefore, the amorphous P in SHS15 and WSS10 is more likely associated with K than in the SHS40 and WSS30 mixtures. It is feasible that K could be associated with Si rather than P. However, thermodynamic equilibrium calculations conducted on the fuels used in this work would indicate that the formation of CaKPO_4_ or KMgPO_4_ is thermodynamically favored over K silicates.^29^

It is also feasible that small amounts of unidentified crystalline Al phosphates may be present in the residual ashes. A study that investigated the molecular environment of P in four different sewage sludge ashes using a combination of XRD, solid-state ^31^P DPMAS-NMR, and XANES showed that XRD consistently underestimated or failed to identify Al phosphates in the ashes compared to the aforementioned methods.^19^ Although the addition of biomass resulted in a clear shift in the type of identified whitlockite phases from mainly Ca_9_(Fe, Al)(PO_4_)_7_ to Ca_9.5_Mg_1–*x*_Fe_*x*_(PO_4_)_7_ or Ca_9_KMg(PO_4_)_7_, a degree of caution should be used in interpreting these changes in the stoichiometric composition. Because of the similarities in crystal structure between whitlockite-type phases, they produce diffractograms that are hard to differentiate. Therefore, assigning an exact stoichiometric composition to these phases is extremely difficult in complex ashes as other crystalline phases and amorphous backgrounds interfere with the identification.

The phosphates identified in the coarse ash of WS^46^ and SH (Ca_5_(PO_4_)OH, CaKPO_4_) were not identified in the ashes from the co-combustion experiments except for a small amount of Ca_5_(PO_4_). In combination with the lack of SH and WS ash particles in the residual ash, it can be concluded that the biomass ash was thoroughly incorporated into the bulk ash matrix. However, this may not have been the case when the SHS15 mixture was combusted at 800 and 950 °C in a macro-TGA.^27^ A significant share of Ca_5_(PO_4_)OH and CaKPO_4_ was identified in the ash in addition to Ca_9_KMg(PO_4_)_7_. Further, individual grains of ash originating from SS and SH were distinguishable in the ash residue with a low degree of sintering. Therefore, some melt formation is likely required to get a more thorough interaction between the ash particles of the individual fuels in the fuel mixture.

The dominance of orthophosphates indicates that the ash transformation reactions of P in these ash mixtures are dominated by substitution reactions primarily involving K^+^, Ca^2+^, Mg^2+^, Fe^2+^, Fe^3+^, and Al^3+^. These substitution mechanisms are expected given that the ash composition of the individual fuels and the fuel mixtures is cation-rich and most P originates from an inorganic association in the SS fuel.^28,52^ Because of the large share of melt in the residual ash fractions, these substitution reactions are likely to have occurred through liquid–liquid interactions. However, the formation of K-containing phosphates may be restricted because of the immiscibility gap in the system, as the partitioning of Si and P may limit the interaction between the two systems. On the basis of the Rhenania process,^55^ substitution reactions between the Ca-rich phosphates and K silicates are likely necessary to facilitate the formation of phosphates with a higher share of alkali.

On the basis of the overall outcomes of the co-combustion experiments, SH is a more suitable co-combustion fuel than WS for improving the recovery potential of P and reducing the risk of ash-related issues. This qualitative assessment considers that formation of K-bearing phosphates is beneficial for P recovery and that formation of K silicate phases may cause severe slagging issues. The lack of CaKPO_4_ and KMgPO_4_ in the residual ash fractions of WSS10 and SHS15 indicates that ash mixtures with an even higher K share are required to favor the formation of phosphates with higher shares of K. However, considering the trend in slag formation for the WSS mixtures, increasing the share of WS past 90 wt % may cause issues in terms of slag formation. The ash composition of the sewage sludge also needs to be considered as the ash composition of sewage sludges can vary significantly.^56^ On the basis of these results, sewage sludges with a high content of Al would likely increase the amount of alkali required to alter the speciation of P toward K-bearing phosphates. Combining co-combustion and alkali additives such as (K, Na)_2_CO_3_ and (K, Na)_2_SO_4_ may be a suitable option to allow for a larger flexibility in biomass fuels used. Future research should investigate a broader range of ash-forming elements by using a variety of sewage sludges, biomasses, and additives. A more holistic approach is needed to determine general guidelines and molar ratios for utilizing compositionally diverse sewage sludge fuels in co-combustion systems for P recovery.

## 4 Conclusions

Co-combustion of sewage sludge (SS) with K-rich sunflower husk (SH) or K- and Si-rich wheat straw (WS) was investigated to determine the ash transformation of P and the formation of slag and fine particulate matter (PM_1_) to improve the P recovery potential of the ash and reduce the risk of ash-related issues. The pure fuels (SS, SH, WSS) and four fuel mixtures were combusted in a residential pellet burner (∼12 kW_th_), and the ashes were collected and characterized using CHN analysis, SEM/EDS, and XRD.

The co-combustion of SS with SH or WS resulted in similar or lower fine particulate matter concentrations compared to the reference experiments with SH and WS. The retention of P in residual ash fractions, i.e., slag and bottom ash, was high for all fuels and fuel mixtures (>97%). The retention of K in the residual ash fractions was high for all fuels (>84%) except for SH, where less than 60% of K was retained in the residual ash.

The co-combustion experiments significantly reduced slag formation compared to the reference experiment with SS. Co-combustion of SS with SH had a higher potential for reducing slag formation than WS as it produced the lowest amount of slag at the higher addition level of biomass. The extensive interaction of main ash-forming elements derived from different individual fuels in the fuel mixture pellets showed that co-combustion successfully changed the ash behavior, representing the composition of the fuel mixture rather than the individual constituent fuels. The spatial distribution of elements and the morphology in the slag cross sections showed that P-rich regions precipitated within the slag matrix. The share of phosphates associated with Fe decreased in favor of Ca as more biomass was added to the mixture. However, the dominating phosphate phases were whitlockites (Ca_(11-*x*-*y*-*z*)_K_*x*_(Ca, Mg, Fe)_*y*_(Fe, Al)_*z*_(PO_4_)_7_) in all residual ash fractions. Amorphous phases rich in P and K were present in the residual ash fractions of the high-biomass mixtures. The results also confirm the tendency of K to be incorporated in K-Al silicates instead of K-bearing phosphates that was seen in previous lab-scale studies.

This experimental study elucidates practically relevant process characteristics for continuous combustion of fuels previously analyzed in single-pellet investigations in a fixed-bed setup. Thus, it was shown that it is possible to significantly alter the speciation of P in sewage sludge ashes through fixed-bed co-combustion with agricultural biomass. However, the outcome of the co-combustion experiments indicates that an even higher relative concentration of K in the ashes is required to produce a high share of K-bearing phosphates. On the basis of the overall behavior of P, co-combustion of sewage sludge with agricultural residues in fixed-bed combustion is a promising option to improve the recovery potential of P in sewage sludges. Furthermore, co-combustion of SS with low-value biomass showed operational benefits such as an improved overall combustion performance and reduced risk of slag formation compared to mono-combustion.
